# Phytohormone and Amino Acid Changes in Cherry Radish as Metabolic Adaptive Response to Arsenic Single and Multi-Contamination

**DOI:** 10.3390/biom15030390

**Published:** 2025-03-08

**Authors:** Daniela Pavlíková, Veronika Zemanová, Milan Pavlík, Marie Lhotská, Jan Kubeš, Milan Novák, Petre I. Dobrev, Václav Motyka

**Affiliations:** 1 Department of Agroenvironmental Chemistry and Plant Nutrition, Faculty of Agrobiology, Food and Natural Resources, Czech University of Life Sciences Prague, 16500 Prague, Czech Republic; pavlik.milan.pm@gmail.com (M.P.);; 2 Czech Agrifood Research Center, Division of Crop Management Systems, 16100 Prague, Czech Republic; 3 Department of Botany and Plant Physiology, Faculty of Agrobiology, Food and Natural Resources, Czech University of Life Sciences Prague, 16500 Prague, Czech Republickubes@af.czu.cz (J.K.); 4 Laboratory of Hormonal Regulations in Plants, Institute of Experimental Botany of the Czech Academy of Sciences, 16502 Prague, Czech Republic

**Keywords:** amino acids, metalloid, hormones, stress, vegetable

## Abstract

This study investigated the metabolic adaptive responses to As contamination and As co-contamination with cadmium, lead, and zinc in the leaves and tubers of cherry radish (*Raphanus sativus* var. *sativus* Pers.). The response was assessed by measuring malondialdehyde levels, total phenolic content (TPC), total anthocyanin pigment (TAC), growth and stress phytohormone concentration, and free amino acid content. The characteristic As accumulation of single contamination resulted in a decrease in tuber growth. However, in the case of co-contamination, As uptake was influenced by the presence of other potentially toxic elements (PTEs), mainly zinc, with no significant effect on growth. Both contaminated treatments exhibited significant differences in metabolite levels among the organs, along with notable changes in their contents. Increases in malondialdehyde, TPC, and TAC indicated induced oxidative stress and an antioxidant response that was more pronounced by As co-contamination. Also, the results for phytohormones, which showed both increases and decreases, along with selected free amino acids (which showed increases), demonstrated a more significant influence of As co-contamination. Based on these findings, it can be concluded that the response of cherry radish to contaminated treatments exhibited significant differences in the studied parameters, along with variability in the results, reflecting the extent of the effects of PTEs that induce oxidative stress.

## 1. Introduction

Anthropogenic activities are the main cause of potentially toxic element (PTE) pollution in the environment, and soil serves as a major natural reservoir for these elements [1,2]. Arsenic (As) is one of the PTEs that occurs naturally in the environment. Due to its high mobility, it can rapidly impact plants and crops [3]. Arsenic is an extremely toxic metalloid to plants, animals, and humans [4]. The As inorganic form is far more toxic than most organic forms found in the environment [5]. Under oxidizing conditions, arsenate—As (V)—is the dominant inorganic form, whereas arsenite—As (III)—is prevalent in reducing conditions. These two forms are interconvertible [6,7]. As (III) is more toxic than As (V), and they differ in both their mechanisms of toxicity and their transport within plants [8].

The level of toxicity varies among plant species classified as bioindicators or normal plants [9]; however, the effects remain largely consistent [10]. Arsenic and other PTEs exhibit physiological, biochemical, and morphological effects on plants [10,11,12]. Similar to other PTEs, As disrupts photosynthesis and transpiration by decreasing photosynthetic pigments and gaseous exchange parameters, ultimately causing leaf senescence [13,14]. The influence of As and other PTEs on plants is typically linked to oxidative stress, which impacts the regulation of various metabolic pathways [3,12,15,16,17]. One of the metabolic pathways affected by As or other PTEs stress is the metabolism of amino acids (AAs), as confirmed by several studies [17,18,19,20,21,22]. Potentially toxic elements, such as As, primarily regulate the homeostasis of stress and growth phytohormones (PHs) through oxidative stress. The stress PHs induce stress metabolism, thus establishing a cross talk between PHs and AAs homeostasis. Amino acids, as key components of membrane proteins and enzymes, function as substrates for the biosynthesis of crucial primary metabolites and PHs [23,24,25]. They also act as precursors for the synthesis of various biologically active secondary metabolites in plants, which can be either toxic or antioxidant [26,27,28].

Plants have various strategies to cope with stress induced by PTEs that involve the coordination of various metabolic pathways [4]. Especially, the metabolism of carbon (C), nitrogen (N), and sulfur is essential for the effective adaptation to stress from PTEs [29,30,31,32]. Within plants, sulfur metabolism plays an essential role in PTEs detoxification, as sulfhydryl groups in peptides like glutathione and phytochelatins bind to As, facilitating the sequestration of these complexes into vacuoles [4,33,34]. According to Awasthi et al. [4], tolerant plant varieties have signaling mechanisms to detect As stress, involving various players such as PHs, reactive oxygen species, nitric oxide, etc.

According to Gopalapillai and Hale [35], most contaminated soils contain elevated levels of multiple PTEs rather than just one, with the combined environmental effects differing from those of individual PTEs. Arsenic can interact with other PTEs such as cadmium (Cd) [36], lead (Pb) [37], and zinc (Zn) [38]. Gong et al. [38] reported that the presence of As and Zn can either mitigate or exacerbate toxicity due to the essentiality of Zn and the non-essential nature of As. While numerous studies have explored the effect of As single contamination on plant metabolism [8,20,21,39,40,41,42], fewer have investigated the impacts of combined As, Cd, Pb, and Zn contamination on plant metabolic processes [17,36,37,38,43,44,45,46]. Therefore, the objective of this study was to compare the effects of As contamination individually versus co-contamination with Cd, Pb, and Zn on plant stress metabolism. The influence of co-contamination with Cd, Pb, and Zn was possible to characterize due to the similar content of As in the experimental soils. For the evaluation of stress response in the experiment, cherry radish was selected. This plant is known for its relatively fast growth [47] and is a popular vegetable commonly cultivated in the Czech Republic. We assumed differences in the response of cherry radish to As single contamination and co-contamination with Cd, Pb, and Zn. Therefore, responses to induced stress were characterized by changes in the content of selected PHs, free AAs, and stress metabolites, along with their interactions. The findings will deepen the knowledge of how As, both in single contamination and co-contamination, influences plant metabolism.

## 2. Materials and Methods

### 2.1. Plant Material and Pot Experiment

A greenhouse pot experiment was conducted using a randomized design with four replications for each treatment. Pots were filled with 2.5 kg of soil sourced from both non-polluted and anthropogenically polluted regions of the Czech Republic (Table 1) based on the experimental setup. The experiment comprised the following treatments: (i) Control—soil without added As; (ii) As—soil spiked with As (20 mg·kg^−1^); and (iii) As+Cd+Pb+Zn—soil with anthropogenic pollution from smelting and lead processing [48]. The Control and As treatments used the same soil, classified as non-polluted according to Czech standards for the content of PTEs in soil (20 mg·kg^−1^ for As, 0.5 mg·kg^−1^ for Cd, 60 mg·kg^−1^ for Pb, and 120 mg·kg^−1^ for Zn). Arsenic was introduced into the As treatment using a Na_2_HAsO_4_ solution, which was thoroughly mixed into the soil. Following a three-month maturation period, the pseudo-total As content in the spiked soil was comparable to that in the As+Cd+Pb+Zn treatment. The soil of each pot was supplemented with 0.5 g N, 0.4 g K, and 0.16 g P (applied as NH_4_NO_3_ and K_2_HPO_4_ solutions per pot).

Cherry radish (*Raphanus sativus* var. *sativus* Pers. ‘Viola’) was selected as the test plant, with seeds (Nohel Garden a.s., purchased from a store in the Czech Republic) sown directly into the soil at a rate of 15 seeds per pot. After the development of two true leaves, thinning was carried out to retain 6 seedlings per pot. The experiment was conducted under semi-controlled conditions, including natural light, day/night temperature of 20–23 °C/15–18 °C, and a relative humidity of ~60%. After 50 days of growth, plants were harvested and divided into roots, tubers, and leaves. Each plant part was washed with distilled water, blotted dry, and weighed. Samples were prepared for further analysis, with one portion frozen in liquid nitrogen and stored at −80 °C for metabolite analysis, while another was oven-dried at 40 °C to a constant weight and homogenized for element analysis.

### 2.2. Determination of Potentially Toxic Elements

The contents of As, Cd, Pb, and Zn were measured using an Agilent 720 inductively coupled plasma optical emission spectrometer (ICP-OES; Agilent Technologies Inc., Santa Clara, CA, USA) following low-pressure microwave digestion with a 4:1 (*v*/*v*) mixture of HNO_3_ and H_2_O_2_ (10 mL; Ethos 1, MLS GmbH,Leutkirch im Allgäu, Germany), as previously described [17,41].

### 2.3. Determination of Phytohormones

Phytohormones in fresh biomass were extracted following established method [49] and analyzed with an LC/MS system consisting of a UHPLC 1290 Infinity II (Agilent Technologies Inc., Santa Clara, CA, USA) coupled with a 6495 Triple Quadrupole Mass Spectrometer (Agilent Technologies Inc.), operating in MRM mode. Quantification was performed using the isotope dilution method, and data acquisition and processing were carried out using Mass Hunter software B.08 (Agilent Technologies Inc.).

### 2.4. Determination of Free Amino Acids

Free AAs in plant biomass were extracted, derivatized, and analyzed following established methods [41]. Derivatization of the extracts was performed using an EZ:faast kit (Phenomenex, Torrance, CA, USA) according to the manufacturer’s protocol. The prepared samples were then analyzed using a Hewlett Packard 6890 N/5975 MSD gas chromatography–mass spectrometry system (GC-MS; Agilent Technologies Inc., Santa Clara, CA, USA) equipped with a ZB-AAA 10 m 0.25 mm AA analysis GC column.

### 2.5. Determination of Malondialdehyde Content

Malondialdehyde (MDA) content was determined using a modified thiobarbituric acid (TBA) method, as previously described [17]. The absorbance of samples at 440 nm, 532 nm, and 600 nm was measured on a UV/VIS spectrophotometer (Evolution 201, Thermo Scientific, Waltham, MA, USA). The MDA content (nmol·g^−1^ FW) was calculated by the equation of Heath and Packer [50].

### 2.6. Determination of Total Phenolic Content

The total phenol content (TPC) was analyzed by an assay slightly adapted by Singleton and Rossi [51]. The extract used for MDA determination was mixed with a 10-fold diluted Folin–Ciocalteu reagent, followed by the addition of 7% sodium carbonate after 5 min of incubation. The sample absorbances were measured in 765 nm after 90 min on a UV/VIS spectrophotometer (Evolution 201, Thermo ScientificWaltham, MA, USA). The TPC was expressed as gallic acid equivalent (mg GAE·g^−1^ FW).

### 2.7. Determination of Anthocyanin Content

The total anthocyanin content (TAC) was determined using pH differential method [52]. Ethanolic extracts were diluted with either potassium chloride buffer (pH 1) or sodium acetate buffer (pH 4.5). The absorbance of solutions was measured after 15 min of incubation at 520 nm for anthocyanin determination and 700 nm for correction of eventual haze using a UV/VIS spectrophotometer (Evolution 201, Thermo Scientific, Waltham, MA, USA). The absorbance was calculated using the formula (A × MW × DF × 1000)/(e × 1), where A represents absorbance, MW is the molecular weight of cyanidin-3-glucoside (449.2 g·mol^−1^), e is the molar extinction coefficient of this anthocyanin (26,900), and DF is dilution factor. A pathlength was 1 cm, and the results were expressed in mg·100 g^−1^, taking into regard the mass of the sample and the volume of the extract solution used.

### 2.8. Statistical Analysis

Statistical analyses were conducted using Statistica 12.0 software (StatSoft, Tulsa, OK, USA) and XLStat 2023.1.3 software (Lumivero, Burlington, MA, USA). Data are presented as the mean values with standard deviation (SD) for four biological replicates per treatment. One-way ANOVA followed by Tukey’s post-hoc test (*p* < 0.05) was applied to determine statistically significant differences among treatments. Pearson’s linear correlation analysis (*p* < 0.05) was also performed to assess relationships between variables.

## 3. Results

### 3.1. Accumulation and Transport of Potentially Toxic Elements in the Cherry Radish Under Single and Co-Contamination and Its Effect on Growth

The results indicated a significant difference in the accumulation of As in relation to the treatment—i.e., As treatment versus As+Cd+Pb+Zn treatment (Table 2). The radish roots in the As treatment exhibited the highest As content, while the content in all plant parts of the Control and the As+Cd+Pb+Zn treatment was below the limit of detection. Arsenic also accumulated in radish tubers; however, the radish failed to translocate As to the leaves. In contrast to As accumulation, radish leaves contained the highest Cd content, suggesting a more substantial translocation of Cd. The results of Pb accumulation followed a pattern similar to that of As accumulation. The highest Pb content was observed in radish roots of the As+Cd+Pb+Zn treatment, while the content in all plant parts of the Control and the As treatment was below the limit of detection. The content of Zn varied among plant parts and treatments. The highest Zn content was found in all radish parts of the As+Cd+Pb+Zn treatment (Table 2), indicating significant Zn translocation to the leaves.

**Figure 1:**
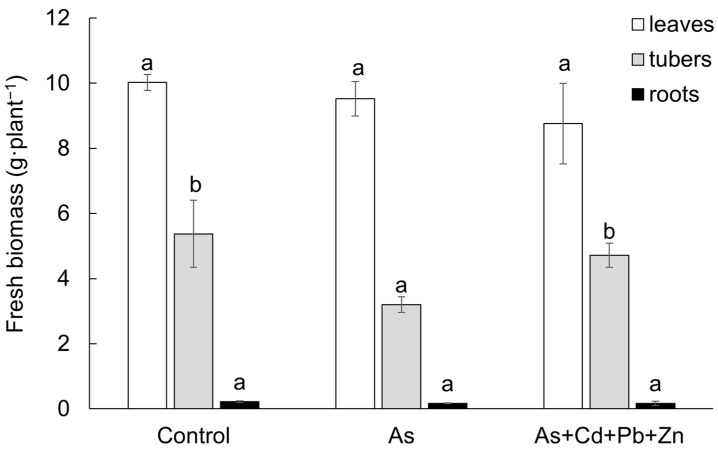
Fresh biomass of the individual parts of cherry radish growing in the soil without contamination (Control), with As contamination (As) and As co-contamination (As+Cd+Pb+Zn) for 50 days. Values represent the mean ± SD (*n* = 4). Data with the same letter are not significantly different. Different letters indicate significant differences among treatments according to the ANOVA with Tukey’s test (*p* ˂ 0.05).

The impact of both contaminations on the growth of cherry radish showed a consistent trend across all plant parts, resulting in a reduction in fresh biomass in comparison to the Control. However, the trend was found to be statistically significant only in the tubers of the As treatment (Figure 1). Specifically, leaf fresh biomass declined by 5% in the As treatment and by 13% in the As+Cd+Pb+Zn treatment when compared to the Control. Similarly, the fresh biomass of tubers was reduced by 40% and 12% in the As and As+Cd+Pb+Zn treatments, respectively. Furthermore, in comparison to the Control, the fresh biomass of roots also declined by 23% and 21% in the As and As+Cd+Pb+Zn treatments, respectively.

### 3.2. Effect of Accumulated Potentially Toxic Elements on Oxidative Stress Reflecting Fitness Status of Cherry Radish Under Single and Co-Contamination

The stress response of cherry radish in both leaves and tubers was evaluated based on the content of MDA and TPC. Additionally, the TAC in the tubers of cherry radish was analyzed as an antioxidative metabolite. Monitoring the regulation of homeostasis concerning MDA, TPC, and TAC is crucial for assessing oxidative stress levels and understanding the mechanisms that reflect the status of the induced antioxidant system in plants.

**Figure 2:**
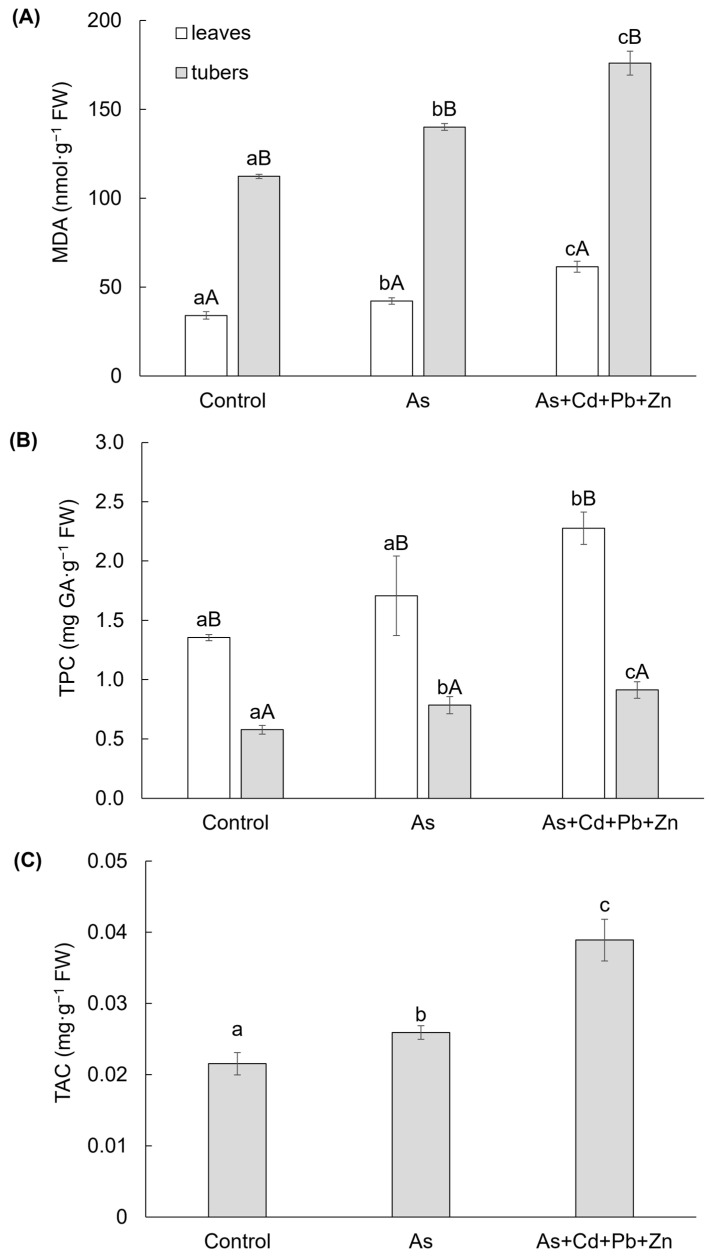
Content of malondialdehyde (**A**), total phenolic (**B**), and total anthocyanin (**C**) in the individual parts of cherry radish growing in the soil without contamination (Control), with As contamination (As) and As co-contamination (As+Cd+Pb+Zn) for 50 days. Data represent the mean ± SD (*n* = 4). Data with the same letter are not significantly different. Different letters indicate significant differences among treatments (lowercase letters) and plant parts (uppercase letters) according to the ANOVA with Tukey’s test (*p* ˂ 0.05).

Both contaminated treatments had an impact on the content of MDA in the leaves and tubers of cherry radish (Figure 2A), which serves as a bioindicator of membrane damage resulting from oxidative stress following the degradation of unsaturated fatty acids. Significantly higher content of MDA was observed in the tubers of cherry radish. In comparison to the Control, the MDA contents increased in the leaves and tubers due to As treatment (by 24% and 25%, respectively) and As+Cd+Pb+Zn treatment (by 80% and 57%, respectively). These findings indicate a greater extent of damage in the tubers and a higher level of toxicity associated with the As+Cd+Pb+Zn treatment.

Significant differences were also observed in the TPC, a group of antioxidant secondary metabolites, in the leaves and tubers of cherry radish (Figure 2B). The TPC was higher in the leaves compared to the tubers. However, the impact of both contaminated treatments was only significant in the tubers. In comparison to the Control, the TPC increased by 36% and 58% in the tubers under As and As+Cd+Pb+Zn treatments, respectively. In the leaves, a significant increase in TPC was observed only in response to the As+Cd+Pb+Zn treatment (by 45%). Additionally, TAC exhibited a significant change due to the As and As+Cd+Pb+Zn treatments (Figure 2C). In the tubers of cherry radish, TAC increased by 20% with As treatment and by 81% with As+Cd+Pb+Zn treatment compared to the Control.

### 3.3. Effect of Accumulated Potentially Toxic Elements on the Regulation of Phytohormone Homeostasis Reflecting Fitness Status of Cherry Radish Under Single and Co-Contamination

**Figure 3:**
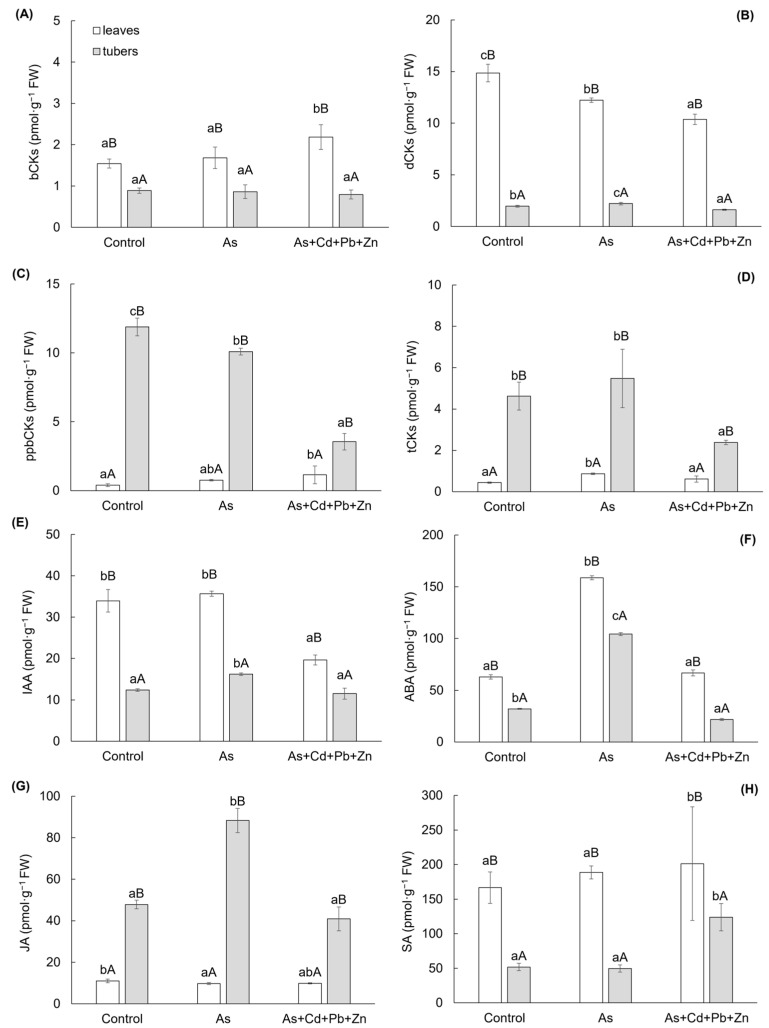
Content of growth phytohormones (**A**–**E**): bioactive forms of cytokinins (**A**), inactive or weakly active forms of cytokinins (**B**), primary products of cytokinin biosynthesis (**C**), transport forms of cytokinins (**D**), indole-3-acetic acid (**E**); and stress phytohormones (**F**–**H**): abscisic acid (**F**), jasmonic acid (**G**), and salicylic acid (**H**) in the individual parts of cherry radish growing in the soil without contamination (Control), with As contamination (As) and As co-contamination (As+Cd+Pb+Zn) for 50 days. Data represent the mean ± SD (*n* = 4). Data with the same letter are not significantly different. Different letters indicate significant differences among treatments (lowercase letters) and plant parts (uppercase letters) according to the ANOVA with Tukey’s test (*p* ˂ 0.05).

The results of growth plant hormones, cytokinins and auxin, exhibited significant variances among cherry radish parts, along with a significant impact of As+Cd+Pb+Zn treatment (Figure 3A–E). The change of determined cytokinins was categorized into five groups based on their structure and physiological functions: (i) bioactive forms (bCKs—i.e., free bases including *trans*-zeatin, *cis*-zeatin, isopentenyl adenine), (ii) inactive or weakly active forms (dCKs—i.e., *N*-glucosieds including *trans*-zeatin-7-glucoside, *cis*-zeatin-9-glucoside, isopentenyl adenine-7-glucoside, isopentenyl adenine-9-glucoside), (iii) primary products of cytokinins biosynthesis (ppbCKs—i.e., nucleotides including *trans*-zeatin riboside monophosphate, *cis*-zeatin riboside monophosphate, isopentenyl adenosine monophosphate), (iv) transport forms (tCKs—i.e., ribosides including *trans*-zeatin-9-riboside, *cis*-zeatin-9-riboside, isopentenyl adenosine), and (v) storage forms (i.e., *O*-glucosides). Within the last group, only *cis*-zeatin riboside-O-glucoside was identified, which constituted 0.4–1% of the total cytokinins content and exhibited an increase with As and As+Cd+Pb+Zn treatment (0.069 ± 0.008 pmol·g^−1^ FW and 0.096 ± 0.014 pmol·g^−1^ FW, respectively) in the leaves compared to the Control (0.047 ± 0.003 pmol·g^−1^ FW). However, the change in content in the tubers was not significant.

Among the different variants, the bCKs and dCKs groups exhibited significantly higher contents in the leaves of cherry radish (Figure 3A–B). The contents of bCKs and dCKs accounted for 9–15% and 72–86% of the total cytokinin content, respectively. On the other hand, the ppbCKs and tCKs groups showed higher contents in the tubers of cherry radish (Figure 3C, D). The contents of ppbCKs and tCKs represented 42–61% and 24–29% of all cytokinin content, respectively. In comparison to the Control, the bCKs group displayed a 41% increase in leaf content with As+Cd+Pb+Zn treatment, while the change in tubers was not statistically significant (Figure 3A). The differences in dCKs content were observed between As and As+Cd+Pb+Zn (Figure 3B), with content decreasing by 18% and 30% in leaves for each treatment, respectively. In tubers, the content increased by 13% with As treatment and decreased by 17% with the As+Cd+Pb+Zn treatment. The ppbCKs group (Figure 3C) exhibited a similar trend in cherry radish tubers as the dCKs group in the leaves. In comparison to the Control, ppbCKs content decreased by 15% with As treatment and by 70% with As+Cd+Pb+Zn treatment. On the other hand, a significant increase was observed in leaves only with the As+Cd+Pb+Zn treatment (by 188%). Additionally, the tCKs group (Figure 3D) in cherry radish tubers showed a significant 48% decrease only with the As+Cd+Pb+Zn treatment. In the leaves of cherry radish, this group increased by 98% with As treatment compared to the Control. The other major group of growth PHs, auxin—indole-3-acetic acid (IAA, Figure 3E)—increased by 30% with As treatment in cherry radish tubers and decreased by 42% with As+Cd+Pb+Zn treatment in the leaves.

Similarly to the growth PHs, the group of stress PHs (abscisic acid—ABA, jasmonic acid—JA, salicylic acid—SA) showed significant differences among the parts of cherry radish (Figure 3F–H). In contrast to the growth PHs, a significant effect on stress PHs was observed with As treatment, except for SA (Figure 3H). Among the mentioned stress PHs, ABA and SA showed significantly higher content in the leaves, while the content of JA was higher in the tubers of cherry radish (Figure 3F–H). In As treatment, ABA content increased by 153% in the leaves and 225% in the tubers of cherry radish compared to the control (Figure 3F). Conversely, under As+Cd+Pb+Zn treatment, a decrease in ABA was observed solely in the tubers. JA content was significantly impacted only by As treatment (Figure 3G), decreasing by 11% in the leaves and increasing by 85% in the tubers compared to the Control. Regarding SA, a significant change was observed only in the As+Cd+Pb+Zn treatment in both organs of cherry radish (Figure 3H), with SA content increasing by 21% in the leaves and 139% in the tubers compared to the Control.

### 3.4. Effect of Accumulated Potentially Toxic Elements on Regulation of Free Amino Acid Homeostasis Reflecting Fitness Status of Cherry Radish Under Single and Co-Contamination

**Figure 4:**
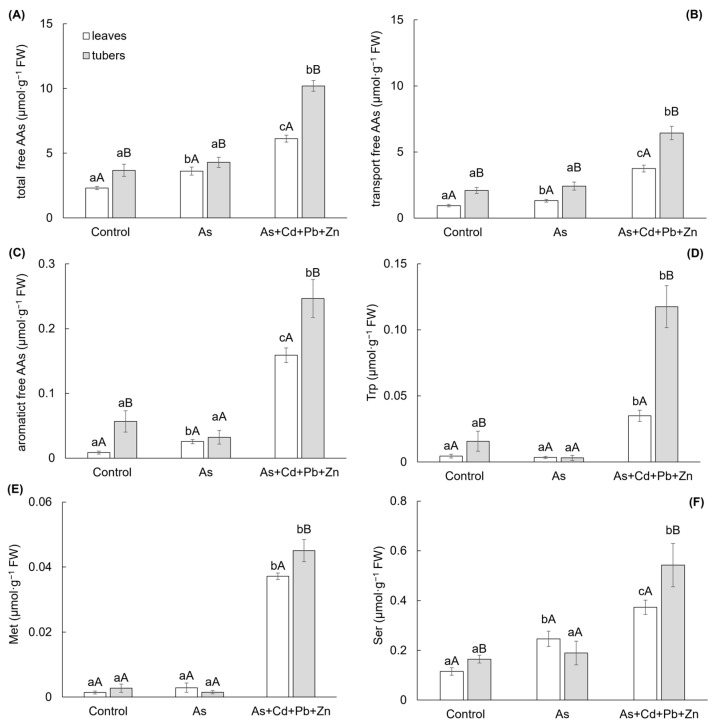
Total content of free amino acids (**A**), transport form of free amino acids (**B**), aromatic free amino acids (**C**), free tryptophan (**D**), free methionine (**E**), and free serine (**F**) in the individual parts of cherry radish growing in the soil without contamination (Control), with As contamination (As) and As co-contamination (As+Cd+Pb+Zn) for 50 days. Values represent the mean ± SD (*n* = 4). Data with the same letter are not significantly different. Different letters indicate significant differences among treatments (lowercase letters) and plant parts (uppercase letters) according to the ANOVA with Tukey’s test (*p* ˂ 0.05).

Changes in the homeostasis of the store of free AAs were monitored because they are a source for the biosynthesis of membrane proteins, enzymes, growth PHs, and last but not least, they are irreplaceable for the formation of a specific compound necessary for the antioxidant system, which serves plants to overcome oxidative stress. Both contaminated treatments affected the metabolism of free AAs in leaves and tubers of cherry radish (Figure 4A–F). Generally, higher contents of free AAs were determined in the tubers among treatments. The total content of free AAs (Figure 4A) increased in leaves by As and As+Cd+Pb+Zn treatments (by 57% and 165%, respectively). On the other hand, the increase of total free AAs in tubers was significant only for As+Cd+Pb+Zn treatment (by 177%).

The main part of the total free AAs content showed a group of transport AAs (glutamic acid, glutamine, aspartic acid, and asparagine), which content reached 36–61% and 56–63% of the total free AAs content in the leaves and tubers, respectively (Figure 4B). The change in this group showed the same trend as the total content of free AAs. Similarly, the content of another group of free AAs—aromatic AAs (tryptophan—Trp, phenylalanine, and tyrosine; Figure 4C) demonstrated an increase in leaves due to As and As+Cd+Pb+Zn treatments (by 198% and 1753%, respectively), with a significant increase observed only in tubers for the As+Cd+Pb+Zn treatment (by 335%). Free Trp was found as the main free AAs of the aromatic AAs group, representing 13–51% and 10–48% of the aromatic AAs content in the leaves and tubers, respectively (Figure 4D). The change in Trp content mirrored those of the aromatic AAs group. A significant increase was determined at As+Cd+Pb+Zn treatment in both leaves and tubers—by 698% and 650%, respectively.

The high toxicity associated with the As+Cd+Pb+Zn treatment indicated a notable increase in the content of free methionine (Met), which exhibited a rise of 2553% and 1563% in the leaves and tubers of cherry radish (Figure 4E). Also, another free AA—free serine (Ser)—showed a significant response to the As+Cd+Pb+Zn treatment, resulting in a 230% increase in Ser content in the tubers of cherry radish (Figure 4F). On the other hand, in the leaves, Ser, as an AAs associated with photorespiration, was influenced by both As treatment and As+Cd+Pb+Zn treatment, leading to increases of 114% and 225%, respectively.

### 3.5. The Relationships Between Metabolites of Cherry Radish Under Single and Co-Contamination

**Figure 5:**
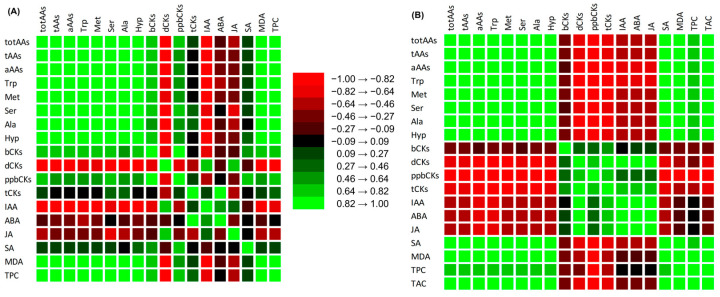
Correlation matrix between metabolites in leaves (**A**) and tubers (**B**) of cherry radish (*p* < 0.05, Table S1).

Among treatments, the relationship between determined metabolites of cherry radish was calculated by correlation (Table S1 and S2) and visualized as a correlation matrix (Figure 5). The results confirmed a significant difference in metabolite regulation between leaves and tubers, irrespective of the treatment. Positive correlations were observed among free AAs in both the leaves and tubers of radish, while the majority of PHs showed negative correlations (Figure 5, Table S1 and S2).

## 4. Discussion

Plants can accumulate As in various quantities depending on factors such as plant species and soil properties [53]. The As content in plants grown in uncontaminated soils typically ranges from 0.01 to 1.5 mg·kg^−1^ dry weight [54]. However, in contaminated soils, the As content in plant biomass can be higher, as demonstrated by our results on the accumulation of As in roots and tubers of cherry radish under As treatment conditions. While As can accumulate to toxic levels in different plant organs [3], the translocation of As from roots to shoots is generally limited, except in the case of As hyperaccumulators [55]. A similar trend of low As translocation, as previously observed in cherry radish [41], was shown in the current experiment under As treatment, where As was only translocated to the tubers and not to the leaves. Pickering et al. [56] reported that phytochelatins bind to As, leading to As accumulation in roots and limiting As transport to aboveground parts of plants by forming As–phytochelatin complexes. Arsenic accumulation in plants can be affected by microbial As methylation in the soil. In this process, S-adenosylmethionine and methylcobalamin serve as methyl donors for As methylation. The increased volatilization of methylated As presents a potential mitigation point for As mobility and toxicity in the environment [57]. Conditions that promote As methylation include an increased activity of the transmethylation cycle, with MET and S-adenosylmethionine being the primary metabolites of this cycle. Plants enhance the transmethylation cycle in bacteria through root exudates that contain the amino acid MET. An increased content of MET in plants is correlated with MDA levels. This process allows to use synthesized thiol-rich peptides, such as glutathione and phytochelatins, for the detoxification PTEs [58].

Accumulation patterns of As in cherry radish under As single contamination were confirmed to be typical. However, our results suggest that As accumulation was altered in the presence of co-contamination with Cd, Pb, and Zn. The As content was below the detection limit, indicating the influence of other toxic elements present in the soil. Previous studies have shown that the uptake of As can be influenced by Cd, Pb, and Zn in *Pteris vittata* [59]. The authors observed reduced As uptake in *P. vittata* when Cd, Pb, and Zn contents in the soil were below 200 mg·kg^−1^. Similarly, Gong et al. [38] reported a reduction in root uptake of As in the presence of Zn or copper, suggesting antagonistic interactions. Application of Zn was found to reduce the As content in wheat grains [45]. On the other hand, As uptake by *Silene vulgaris* was unaffected by Cd [60]. In the case of cherry radish co-contamination, the change in As accumulation is likely attributed to the presence of Zn in the soil. Antagonistic interaction between As and Zn in soil was reported by Das et al. [61,62]. According to Craw and Chappell [63], the decrease in As contents due to Zn application may be due to the precipitation or fixation of As as Zn-arsenate, rendering it unavailable to plants.

The accumulation of As and other PTEs significantly impacts plant physiology, especially affecting plant biomass [64,65]. Numerous studies have reported reduced growth and yield due to PTE contamination [17,41,66,67,68]. In this study, the fresh biomass of cherry radish did not exhibit significant differences among treatments, except for the tubers of As treatment, which showed a decrease under As stress. Change in growth reflected the accumulation and translocation of PTEs in cherry radish in relation to both single and co-contamination. Despite lack of significant physiological responses, PTEs influenced the metabolism of cherry radish due to the oxidative stress linked to their accumulation [3,12,15,16]. Oxidative stress in both leaves and tubers was indicated by an increase of MDA contents. This marker, used to determine the degree of damage to plants under stress, not only increased with the level of damage but also represented the acclimation process [69,70,71]. Lipid peroxidation is induced by oxidative stress, resulting in the accumulation of MDA and indicating the extent of cellular damage. The similar results observed in both roots and leaves across different plant species demonstrate that PTE stress induces the accumulation of reactive oxygen species (ROS) throughout the entire plant [72]. MDA contents were notably higher in the tubers of cherry radish, reflecting both As single and co-contamination. A similar effect was observed for TPC in the tubers, while the leaves were significantly affected only by the As+Cd+Pb+Zn treatment. Saeed et al. [73] published the significant effect of single metal and combined metal contamination on TPC content. Phenolic compounds play a crucial role in plant defense responses to PTE stress; they mitigate stress-induced changes in plants and modulate the ROS signaling cascade. The importance of phenolic compounds is further underscored by their high chelating capacity for PTEs, which induces oxidative stress, as well as their protective effects on cells against the detrimental impacts of these PTEs [74]. The content of phenols in plant tissues can be used as an indicator of stress [75]. The last marker used to assess the status of the induced antioxidant system in cherry radish was TAC. This antioxidant metabolite increased in the tubers due to both As single and co-contamination. Our findings indicate the activation of the antioxidant plant system in cherry radish and the acclimation process in response to PTE stress. The increased accumulation of anthocyanins during the stress enhances the scavenging of endogenous ROS levels, thereby playing a crucial role in abiotic stress tolerance [76,77]. According to Shoeva and Khlestkina [78], the activation of genes that induce plant anthocyanin synthesis is dependent on the concentration of PTEs. The dependence of this regulatory process to PTEs content in plants is attributed to the strong oxidative stress caused by high PTE concentrations, which subsequently inhibits the regulation of anthocyanin biosynthesis.

The contamination of plants by PTEs significantly alters the hormonal levels, which play a crucial role in regulating stress within plant metabolism [79]. Plant growth hormones primarily support the growth of individual plant parts by regulating changes in homeostasis. In the case of leaves, they also contribute to delaying leaf senescence. Conversely, the homeostasis of stress hormones reflects the extent of harm to the photosynthetic apparatus and the peroxidation of unsaturated fatty acids in cell membranes. Our results of phytohormones showed different effects of As single treatment compared to As co-contamination. ABA plays a significant role against abiotic stress [80]. The internal levels of ABA in plants enhance signaling pathways and activate gene expression [81]. The modulation of ABA biosynthetic genes occurs due to plant exposure to PTEs, consequently stimulating the concentration of ABA within the plants [82]. Its content increases in response to PTE stress, helping to regulate their transport and accumulation [83]. This finding was confirmed by the results of As treatment. Cytokinins play a crucial role in controlling adaptive responses by modifying antioxidant defense systems, maintaining ion balance, and regulating the expression of stress-responsive genes [83]. The content of CKs is increased during tuberous root development, and the IAA/CK ratio may be a key factor in tuberization [84]. The decline in CK contents may result from PTE-induced oxidative stress, leading to the oxidative degradation of these phytohormones [85]. Mohan et al. [86] confirmed the significance of CKs in the elimination of As toxicity, demonstrating a linear relationship between the upregulation of CK oxidase/dehydrogenase (*CKX1*) and the downregulation of CK levels. This process promotes the accumulation of glutathione and phytochelatins. The expression of multiple *CKX* genes is induced by CK, establishing a feedback mechanism that regulates CK activity [87]. The increased expression of *CKX* genes reduces CK content, which causes the enhancement in root growth. The group of inactive or very low active CK nucleotides, referred to as ppbCKs, was shown to decrease in radish due to As content [41]. This finding was confirmed by the decreased contents of ppbCKs in the tubers of As and As+Cd+Pb+Zn treatments, in contrast to the Control in the current experiment. PTE stress leads to the translocation of CKs from the roots to different parts of the plants [80]. Our experiment showed increased contents of bCKs and dCKs in the leaves. The increase in ABA and bCKs contents in the leaves is a response to PTE toxicity and indicates a cross talk between ABA and bCKs signaling pathways [88]. PTE stress affected CK receptor kinases, specifically *AHK2* and *AHK3*, having a negative effect on ABA content and the expression of genes responsible for osmotic stress [89]. bCKs act as ABA antagonists, increasing stomatal conductance and modulating leaf gas exchange [90].

Jasmonates are among the most important plant hormones involved in stress responses. Their key representative, JA, serves as a signaling molecule in numerous processes related to plant metabolism [91]. JA exhibits high tuber-inducing activity and promotes tuber formation, and its levels increased during the early stages of tuber development, specifically during tuber initiation [92]. Our results demonstrate a significant increase in JA content in tubers under the As treatment compared to the Control and As+Cd+Pb+Zn treatments. The enhanced synthesis of JA in plants exposed to As stress promotes the expression of various signaling and stress-responsive genes, which can be upregulated in both roots and leaves. This may explain the role of JA signaling in helping plants cope with stress [93]. Auxin IAA promotes cell division, root and stem growth, and vascular tissue formation, while also enhancing plant nutrient uptake and accumulation. IAA biosynthesis and transport in the roots are inhibited by PTE stress, including Cd and As. A reduction in IAA accumulation in meristematic tissue leads to decreased root growth [85,94]. The decreased IAA content was confirmed in the leaves of As+Cd+Pb+Zn treatment. No significant differences in tuber development were observed in the current experiment. The interaction between JA and IAA mediates root growth inhibition [95]. In our experiment, we found an increased content of SA in both parts of radish grown under As co-contamination treatment. SA is a plant hormone that plays a crucial role in inducing resistance genes and mediating the defense response. It can regulate the transmembrane transport of ions and limit the absorption and transport of toxic elements [96]. A study by Raza and Shafiq [97] demonstrated that SA treatment could significantly reduce Cd accumulation in radish plants. The significantly higher content of SA was observed in the As co-contamination treatment compared to the Control. The accumulation of SA is one of numerous mechanisms in plant metabolism that provides defense against various stresses. The elevated SA content under PTE stress indicates its role as an antioxidant, capable of scavenging ROS and/or indirectly regulating redox balance through the activation of antioxidant responses [98].

Pools of free AAs in plants may change in response to abiotic oxidative stress. Changes in AA content are significant not only for protein biosynthesis but also for other metabolic pathways and signal transduction processes [99]. In our experiment, a strong effect of multicontamination on AA metabolism was confirmed. The total content of free AAs increased in radish leaves grown under both contaminated treatments. These results are consistent with findings for tomato [21] and spinach [18] cultivated in As-contaminated soil. A significant increase in AAs in tubers, in contrast to Control, was observed in the As+Cd+Pb+Zn treatment. Similar results were reported by Pavlíková et al. [41] for carrot, lettuce, and radish exposed to soil pollution from multiple PTEs. The accumulation of free AAs in plants may result from their reduced degradation or from the hydrolysis of proteins during plant stress responses [21].

In accordance with other studies [99,100], the largest part of the total free AAs content was comprised of a group of transport AAs, including glutamic acid, glutamine, aspartic acid, and asparagine, which are primary products of N assimilation. A significant increase in radish leaves was observed across treatments for this group, as well as for a group of aromatic AAs. The pathways for plant aromatic AAs link central C metabolism with the production of L-phenylalanine, L-tyrosine, and L-tryptophan, which are precursors for many specialized aromatic metabolites in plants. During carbohydrate starvation or senescence, the pools of free AAs shift, with aromatic AAs increasing as efficient alternative respiratory substrates [101]. This observation was supported by our results for the As+Cd+Pb+Zn treatment. Tryptophan plays a crucial role as a precursor for the family of auxin hormones [102]. The results for the As+Cd+Pb+Zn treatment showed a decrease in IAA content derived from accumulated Trp. This suggests that the metabolic pathway of auxin biosynthesis, which is derived from Trp, is associated with oxidative stress [103]. In contrast to the co-contamination, the growth of radish tubers in As single contamination did not receive sufficient assimilates due to a significant decrease in aromatic AAs, which are substrates for antioxidant metabolites such as tocopherols and phenylpropanoid compounds. According to Jiang et al. [104], Trp mitigated oxidative damage induced by Cd in broccoli by inhibiting Cd-induced IAA conjugation, ensuring an adequate supply of free IAA to support growth. Trp also enhanced the activity of ascorbate peroxidase under Cd stress.

In our experiment, the high toxicity of the As+Cd+Pb+Zn treatment resulted in a significant increase in free Met content. Methionine, a sulfur-containing AA, is essential for initiating mRNA translation and indirectly regulates various cellular processes. Met serves as a precursor for S-adenosylmethionine (SAM), the primary donor of methyl groups for numerous biological processes, including DNA and protein methylation. SAM functions as a methyl donor in transmethylation reactions and is also a precursor for the synthesis of ethylene, metal ion-chelating compounds, nicotinamide, and phytosiderophores [105].

Plants growth, driven by cell division and elongation, depends on a constant supply of Ser for protein biosynthesis, purine bases for nucleic acid synthesis, phospholipids and sphingolipids for cellular membrane synthesis, glutathione, and auxin. Auxin, a key phytohormone, regulates cell proliferation, ensuring proper growth [106]. Serine, as an AA associated with photorespiration, forms the side chain of Trp, which is a biosynthetic precursor of IAA [107]. Pavlíková et al. [108] reported that the free Ser content was not affected by Zn soil contamination at 250 mg·kg^−1^; however, higher Zn soil contaminations significantly decreased Ser content. Our experiment revealed that the free Ser content in radish grown in As single contamination did not differ from the Control, while our results from the co-contamination showed an opposite trend.

## 5. Conclusions

In this study, we examined the effects of As single and co-contamination on radish metabolism. Monitoring changes in MDA, TPC, and TAC, crucial for assessing oxidative stress levels, confirmed the significant impact of both contaminated treatments on these markers in radish tubers. Our results demonstrated that the contamination of plants with PTEs significantly alters the hormonal levels, which play a critical role in regulating stress within plant metabolism. The observed decline in ppbCK contents in tubers of both contaminated treatments may be attributed to PTE-induced oxidative stress, leading to the oxidative degradation of these phytohormones. The changes in other hormones revealed distinct effects of As single contamination compared to As co-contamination. Arsenic contamination induced increased ABA, IAA, and JA contents in radish, reflecting the adaptive response of plants to As toxicity. The significant increase in SA content in the As-co-contaminated plants enhanced resistance by activating stress-signaling hormonal pathways and overexpressing stress-related genes and enzymes. Notably, the substantial increases in total free, transport, and aromatic AA pools, as well as individual selected AAs in both radish organs, indicated a strong effect of co-contamination on the functional roles of AAs, not only in protein biosynthesis but also in other metabolic pathways. Furthermore, As single contamination caused significant changes in the leaves.

## Figures and Tables

**Table 1:** Basic parameters of experimental soils.

Parameters	Control	As	As+Cd+Pb+Zn
Soil type and subtypepH_H2O_Cation Exchange Capacity (mmol_(+)_·kg^−1^)	Chernozem Haplic	Chernozem Haplic	Cambisol Haplic
	7.5	7.1	6.0
	230.1 ± 5.0	230.1 ± 5.0	165.8 ± 15.1
Total Carbon (%)	2.0 ± 0.08	2.0 ± 0.08	2.4 ± 0.04
As_pseudo-total_ (mg·kg^−1^)	19.76 ± 0.79	40.37 ± 1.13	41.88 ± 1.78
Cd_pseudo-total_ (mg·kg^−1^)	0.39 ± 0.03	0.37 ± 0.01	2.39 ± 0.27
Pb_pseudo-total_ (mg·kg^−1^)	38.95 ± 1.59	37.43 ± 1.38	504.35 ± 20.10
Zn_pseudo-total_ (mg·kg^−1^)	102.21 ± 2.29	98.51 ± 2.96	134.92 ± 0.89

**Table 2:** Content of As, Cd, Pb, and Zn (mg·kg^−1^ dry weight—DW) in the individual parts of cherry radish growing in the soil without contamination (Control), with As contamination (As) and As co-contamination (As+Cd+Pb+Zn) for 50 days. Data represent the mean ± SD (*n* = 4). Data with the same letter are not significantly different. Different letters indicate significant differences among treatments (lowercase letters) and plant parts (uppercase letters) according to the ANOVA with Tukey’s test (*p* ˂ 0.05). nd—values under the limit of detection (As ˂ 3 mg·kg^−1^ DW, Cd ˂ 0.1 mg·kg^−1^ DW, Pb ˂ 2 mg·kg^−1^ DW).

			Content (mg·kg^−1^ DW)	
		Control	As	As+Cd+Pb+Zn
leaves	As	nd	nd	nd
	Cd	0.60 ± 0.05 ^aB^	0.57± 0.13 ^aB^	8.85 ± 1.11 ^bC^
	Pb	nd	nd	15.63 ± 1.66 ^A^
	Zn	34.56 ± 2.08 ^aA^	27.42 ± 2.71 ^aB^	108.19 ± 8.71 ^bB^
tubers	As	nd	4.97 ± 1.15 ^A^	nd
	Cd	0.24 ± 0.07 ^aA^	0.30 ± 0.08 ^aA^	3.96 ± 0.36 ^bA^
	Pb	nd	nd	29.95 ± 9.55 ^B^
	Zn	38.34 ± 13.01 ^bA^	20.13 ± 0.82 ^aA^	58.19 ± 7.49 ^cA^
roots	As	nd	13.90 ± 2.74 ^B^	nd
	Cd	0.35 ± 0.08 ^aA^	0.50 ± 0.13 ^aAB^	5.71 ± 0.82 ^bB^
	Pb	nd	nd	161.13 ± 6.17 ^C^
	Zn	33.17 ± 0.71 ^aA^	30.82 ± 3.63 ^aB^	69.86 ± 0.21 ^bA^

## Data Availability

The data presented in this study are available in the article.

## Supplementary Materials

The following supporting information can be downloaded at https://www.mdpi.com/article/10.3390/biom15030390/s1: Table S1: Correlation coefficients of metabolites in the leaves of radish; Table S2: Correlation coefficients of metabolites in the roots of radish.
