# Supplementary material for: Phytohormone and Amino Acid Changes in Cherry Radish as Metabolic Adaptive Response to Arsenic Single and Multi-Contamination
Source: Biomolecules. 2025 Mar 8;15(3):390. doi: 10.3390/biom15030390 (PMC11940314; doi:10.3390/biom15030390)
Supplement: Supplementary file 1 [file biomolecules-15-00390-s001.zip › biomolecules-3384979-supplementary.pdf]

Table S1 Correlation coefficients of metabolites in the leaves of radish. Values in bold are different from 0 with a significance level $p<0.05$.

Variables	totAAs	tAAs	aAAs	Trp	Met	Ser	Ala	Hyp	bCKs	dCKs	ppbCKs	tCKs	IAA	ABA	JA	SA	MDA	TPC
totAAs		**0.97**	**0.97**	**0.93**	**0.94**	**0.98**	**0.95**	**0.97**	**0.74**	**-0.93**	**0.66**	0.23	**-0.88**	-0.14	-0.56	0.27	**0.97**	**0.87**
tAAs	**0.97**		**1.00**	**0.99**	**0.99**	**0.91**	**0.94**	**0.99**	**0.77**	**-0.84**	**0.61**	0.04	**-0.96**	-0.35	-0.40	0.23	**0.96**	**0.87**
aAAs	**0.97**	**1.00**		**0.99**	**0.99**	**0.90**	**0.93**	**0.99**	**0.77**	**-0.84**	**0.61**	0.03	**-0.96**	-0.37	-0.41	0.22	**0.96**	**0.87**
Trp	**0.93**	**0.99**	**0.99**		**0.99**	**0.84**	**0.92**	**0.97**	**0.73**	**-0.77**	0.55	-0.07	**-0.98**	-0.49	-0.34	0.15	**0.93**	**0.84**
Met	**0.94**	**0.99**	**0.99**	**0.99**		**0.86**	**0.90**	**0.99**	**0.78**	**-0.80**	**0.60**	-0.05	**-0.97**	-0.43	-0.35	0.24	**0.95**	**0.86**
Ser	**0.98**	**0.91**	**0.90**	**0.84**	**0.86**		**0.93**	**0.91**	**0.69**	**-0.96**	**0.66**	0.39	**-0.79**	0.05	**-0.64**	0.26	**0.92**	**0.85**
Ala	**0.95**	**0.94**	**0.93**	**0.92**	**0.90**	**0.93**		**0.92**	**0.63**	**-0.88**	0.52	0.27	**-0.86**	-0.18	-0.53	0.06	**0.92**	**0.86**
Hyp	**0.97**	**0.99**	**0.99**	**0.97**	**0.99**	**0.91**	**0.92**		**0.76**	**-0.85**	**0.63**	0.04	**-0.95**	-0.34	-0.41	0.27	**0.96**	**0.83**
bCKs	**0.74**	**0.77**	**0.77**	**0.73**	**0.78**	**0.69**	**0.63**	**0.76**		**-0.65**	**0.85**	0.02	**-0.75**	-0.21	-0.20	**0.61**	**0.76**	**0.81**
dCKs	**-0.93**	**-0.84**	**-0.84**	**-0.77**	**-0.80**	**-0.96**	**-0.88**	**-0.85**	**-0.65**		**-0.67**	-0.48	**0.73**	-0.14	**0.78**	-0.20	**-0.89**	**-0.85**
ppbCKs	**0.66**	**0.61**	**0.61**	0.55	**0.60**	**0.66**	0.52	**0.63**	**0.85**	**-0.67**		0.17	-0.56	0.04	-0.38	**0.76**	0.57	**0.65**
tCKs	0.23	0.04	0.03	-0.07	-0.05	0.39	0.27	0.04	0.02	-0.48	0.17		0.16	**0.84**	**-0.60**	-0.09	0.18	0.31
IAA	**-0.88**	**-0.96**	**-0.96**	**-0.98**	**-0.97**	**-0.79**	**-0.86**	**-0.95**	**-0.75**	**0.73**	-0.56	0.16		0.54	0.31	-0.15	**-0.90**	**-0.79**
ABA	-0.14	-0.35	-0.37	-0.49	-0.43	0.05	-0.18	-0.34	-0.21	-0.14	0.04	**0.84**	0.54		-0.43	0.08	-0.19	-0.09
JA	-0.56	-0.40	-0.41	-0.34	-0.35	**-0.64**	-0.53	-0.41	-0.20	**0.78**	-0.38	**-0.60**	0.31	-0.43		0.05	-0.51	-0.47
SA	0.27	0.23	0.22	0.15	0.24	0.26	0.06	0.27	**0.61**	-0.20	**0.76**	-0.09	-0.15	0.08	0.05		0.17	0.22
MDA	**0.97**	**0.96**	**0.96**	**0.93**	**0.95**	**0.92**	**0.92**	**0.96**	**0.76**	**-0.89**	0.57	0.18	**-0.90**	-0.19	-0.51	0.17		**0.89**
TPC	**0.87**	**0.87**	**0.87**	**0.84**	**0.86**	**0.85**	**0.86**	**0.83**	**0.81**	**-0.85**	**0.65**	0.31	**-0.79**	-0.09	-0.47	0.22	**0.89**	

Table S2 Correlation coefficients of metabolites in the roots of radish. Values in bold are different from 0 with a significance level $p<0.05$.

Variables	totAAs	tAAs	aAAs	Trp	Met	Ser	Ala	Hyp	bCKs	dCKs	ppbCKs	tCKs	IAA	ABA	JA	SA	MDA	TPC	TAC
totAAs		**0.98**	**0.98**	**0.98**	**0.99**	**0.98**	**0.99**	**1.00**	-0.31	**-0.82**	**-0.98**	**-0.79**	-0.55	-0.52	-0.53	**0.93**	**0.91**	**0.78**	**0.97**
tAAs	**0.98**		**0.95**	**0.95**	**0.97**	**0.93**	**0.97**	**0.97**	-0.36	**-0.80**	**-0.98**	**-0.78**	-0.53	-0.53	-0.55	**0.96**	**0.91**	**0.77**	**0.96**
aAAs	**0.98**	**0.95**		**1.00**	**0.99**	**0.98**	**0.98**	**0.98**	-0.26	**-0.89**	**-0.92**	**-0.82**	**-0.69**	**-0.67**	**-0.66**	**0.91**	**0.82**	**0.67**	**0.91**
Trp	**0.98**	**0.95**	**1.00**		**0.99**	**0.98**	**0.99**	**0.98**	-0.28	**-0.90**	**-0.92**	**-0.84**	**-0.68**	**-0.66**	**-0.66**	**0.91**	**0.83**	**0.68**	**0.91**
Met	**0.99**	**0.97**	**0.99**	**0.99**		**0.98**	**0.99**	**0.99**	-0.31	**-0.87**	**-0.96**	**-0.82**	**-0.63**	**-0.61**	**-0.61**	**0.94**	**0.87**	**0.72**	**0.94**
Ser	**0.98**	**0.93**	**0.98**	**0.98**	**0.98**		**0.98**	**0.99**	-0.23	**-0.81**	**-0.93**	**-0.74**	-0.57	-0.53	-0.52	**0.86**	**0.87**	**0.74**	**0.93**
Ala	**0.99**	**0.97**	**0.98**	**0.99**	**0.99**	**0.98**		**0.99**	-0.29	**-0.84**	**-0.96**	**-0.82**	**-0.59**	-0.55	-0.54	**0.92**	**0.90**	**0.76**	**0.95**
Hyp	**1.00**	**0.97**	**0.98**	**0.98**	**0.99**	**0.99**	**0.99**		-0.30	**-0.80**	**-0.97**	**-0.76**	-0.53	-0.51	-0.51	**0.91**	**0.91**	**0.79**	**0.97**
bCKs	-0.31	-0.36	-0.26	-0.28	-0.31	-0.23	-0.29	-0.30		0.35	0.33	0.50	-0.07	0.11	0.26	-0.33	-0.36	-0.44	-0.37
dCKs	**-0.82**	**-0.80**	**-0.89**	**-0.90**	**-0.87**	**-0.81**	**-0.84**	**-0.80**	0.35		**0.75**	**0.93**	**0.83**	**0.83**	**0.84**	**-0.79**	**-0.58**	-0.41	**-0.70**
ppbCKs	**-0.98**	**-0.98**	**-0.92**	**-0.92**	**-0.96**	**-0.93**	**-0.96**	**-0.97**	0.33	**0.75**		**0.76**	0.44	0.42	0.43	**-0.96**	**-0.96**	**-0.83**	**-0.96**
tCKs	**-0.79**	**-0.78**	**-0.82**	**-0.84**	**-0.82**	**-0.74**	**-0.82**	**-0.76**	0.50	**0.93**	**0.76**		**0.66**	**0.67**	**0.70**	**-0.78**	**-0.64**	-0.50	**-0.72**
IAA	-0.55	-0.53	**-0.69**	**-0.68**	**-0.63**	-0.57	**-0.59**	-0.53	-0.07	**0.83**	0.44	**0.66**		**0.95**	**0.88**	**-0.58**	-0.23	0.03	-0.36
ABA	-0.52	-0.53	**-0.67**	**-0.66**	**-0.61**	-0.53	-0.55	-0.51	0.11	**0.83**	0.42	**0.67**	**0.95**		**0.98**	**-0.59**	-0.19	0.02	-0.37
JA	-0.53	-0.55	**-0.66**	**-0.66**	**-0.61**	-0.52	-0.54	-0.51	0.26	**0.84**	0.43	**0.70**	**0.88**	**0.98**		**-0.61**	-0.21	-0.03	-0.39
SA	**0.93**	**0.96**	**0.91**	**0.91**	**0.94**	**0.86**	**0.92**	**0.91**	-0.33	**-0.79**	**-0.96**	**-0.78**	**-0.58**	**-0.59**	**-0.61**		**0.87**	**0.66**	**0.88**
MDA	**0.91**	**0.91**	**0.82**	**0.83**	**0.87**	**0.87**	**0.90**	**0.91**	-0.36	**-0.58**	**-0.96**	**-0.64**	-0.23	-0.19	-0.21	**0.87**		**0.90**	**0.93**
TPC	**0.78**	**0.77**	**0.67**	**0.68**	**0.72**	**0.74**	**0.76**	**0.79**	-0.44	-0.41	**-0.83**	-0.50	0.03	0.02	-0.03	**0.66**	**0.90**		**0.88**
TAP	**0.97**	**0.96**	**0.91**	**0.91**	**0.94**	**0.93**	**0.95**	**0.97**	-0.37	**-0.70**	**-0.96**	**-0.72**	-0.36	-0.37	-0.39	**0.88**	**0.93**	**0.88**	
